# Paraneoplastic Dermatomyositis presenting as Supraglottitis and acute heart failure; a case report

**DOI:** 10.1093/omcr/omaf167

**Published:** 2025-09-28

**Authors:** John P McCormick, Aiden Kenny, Mark Coyle, Caitlin Waters, Oisin Galvin, Emiliano Bianchini, Faisal Sharif

**Affiliations:** Department of Cardiology, Galway University Hospital, Newcastle Road, Galway, H91 YR71, Ireland; Department of Cardiology, Galway University Hospital, Newcastle Road, Galway, H91 YR71, Ireland; Department of Cardiology, Galway University Hospital, Newcastle Road, Galway, H91 YR71, Ireland; Department of Otolaryngology, Galway University Hospital, Newcastle Road, Galway, H91 YR71, Ireland; Department of Cardiology, Galway University Hospital, Newcastle Road, Galway, H91 YR71, Ireland; Department of Cardiology, Galway University Hospital, Newcastle Road, Galway, H91 YR71, Ireland; Department of Cardiology, Galway University Hospital, Newcastle Road, Galway, H91 YR71, Ireland; School of Medicine, University of Galway, University Road, Galway, H91 TK33, Ireland

**Keywords:** paraneoplastic Dermatomyositis, Supraglottitis, myocarditis, oesophageal carcinoma

## Abstract

Dermatomyositis is a rare autoimmune condition with a highly variable clinical presentation characterized by skin changes and skeletal myopathy. It is frequently associated with underlying malignancy. Rare manifestations including myocarditis and upper airway oedema have been described. We present an unusual case of paraneoplastic dermatomyositis which manifested as acute airway compromise and heart failure in a previously healthy 77-year-old man. The combination of symmetrical proximal myopathy, dysphagia, rash and myocarditis suggested inflammatory myositis. The clinical diagnosis was supported by positive laboratory findings including anti-TIF1 antibodies and characteristic changes on cardiac and musculoskeletal MRI. Endoscopic oesophageal biopsy demonstrated high-grade adenocarcinoma. To our knowledge this is the first reported case of acute supraglottitis as a presentation of paraneoplastic dermatomyositis. Dermatomyositis should be considered in the differential of all patients presenting with muscle weakness and rash, even if other symptoms are dominant at the time of presentation.

## Introduction

Dermatomyositis is a form of idiopathic inflammatory myopathy (IIM) characterized by skin changes and skeletal myopathy. Up to one in four patients with IIM will ultimately be diagnosed with cancer-associated myositis (CAM), in which cancer is diagnosed within three years of IIM onset [1]. While ovarian, lung, pancreatic and stomach are the most frequently associated cancer types; rare cases of IIM related to oesophageal adenocarcinoma have been reported [2]. The 2017 EULAR/ACR criteria for diagnosis of IIM are commonly used in combination with supportive serological and imaging findings [3]. Myocardial and upper airway manifestations of dermatomyositis are extremely rare. This case highlights the highly variable clinical presentation of this rare condition. To our knowledge, it is the first reported case of supraglottitis as the presenting feature of CAM.

## Case report

A 77-year-old Caucasian male presented to the emergency department with acute respiratory distress following two weeks of sore throat and vocal changes. He had attended the emergency department a week prior and had received oral prednisolone and amoxicillin, which he had stopped because of an erythematous rash affecting his face, upper back, shoulders and upper chest in a V-shaped pattern. He also reported a two-month history of fatigue, weakness and unexplained weight loss.

Initial physical examination revealed respiratory distress with stridor. Proximal myopathy affecting the shoulders, neck and hips was noted. Flexible nasoendoscopy demonstrated oedema of the upper airway with reduced vocal cord movement. Laboratory investigations are shown in Table 1. The initial working diagnosis was acute supraglottitis. The patient was admitted to the intensive care unit for airway observation and treated with intravenous antibiotics and corticosteroids.

**Table 1:** Selected laboratory values.

	Normal Range	Day 1	Day 3	Day 7	Day 10	Day 14	Day 21	Day 28
WCC	4–10 ×10^9^/l	4.9	7.9	13.9	15.5	9.6	4.4	6.4
Hemoglobin	13–17 g/dl	12.7	12.7	12.3	12.7	11.9	11.0	12.0
Platelets	150–400 ×10^9^/l	170	211	175	174	145	101	281
CRP	0–5 mg/l	14.4	5.8	5.8	20.3	56	38.7	9.7
ALT	0–40 U/l	31	34	45	40	84	51	35
Creatinine	64–104 umol/L	111	102	130	127	191	134	121
Troponin T	0–14 ng/l	192		329	300	447	475	
Troponin I	0–19.8 ng/l						20	
NT-proBNP	0–400 ng/l	424	701	5153		14 336	1137	569

Over the following days he developed rapidly conducted atrial fibrillation and pulmonary oedema with markedly elevated NT-proBNP levels despite high-dose intravenous diuretics and beta blockers. His vasopressor requirements increased, and he required several emergency cardioversions despite intravenous amiodarone. Transthoracic echocardiography was unremarkable. Cardiac troponin T levels were consistently elevated between 300–800 ng/l (normal values 0–14) while troponin I levels were minimally elevated at 20 (0–19). This raised the possibility of a diffuse myositis, and a comprehensive serological panel was sent. A positive Anti-TIF1 titer was detected.

**Figure 1:**
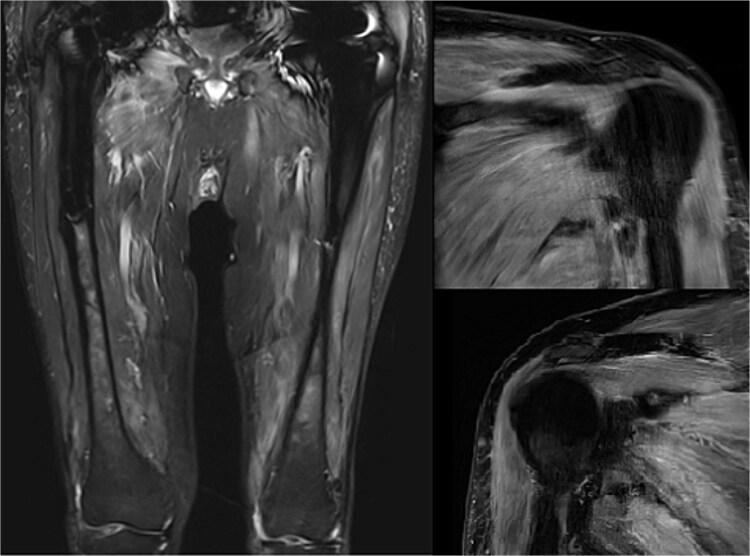
T2 STIR magnetic resonance imaging sequences of the shoulders and femurs demonstrating increased signal intensity. These findings are consistent with acute inflammatory myopathy as opposed to steroid associated myopathy, in which muscle atrophy and fatty infiltration with hyperintensity on T1-weighted sequences would be expected.

**Figure 2:**
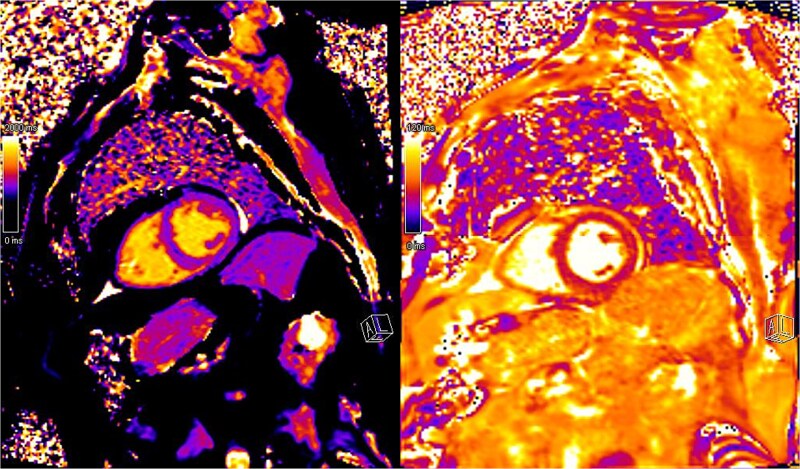
Cardiac magnetic resonance imaging showing elevated T1 (left) and T2 (right) mapping values suggestive of recent myocarditis. There was no evidence of inducible ischemia or recent infarction to explain the elevated cardiac troponin levels.

A provisional diagnosis of dermatomyositis was made based on the patient’s proximal muscle weakness and recent history of typical rash. MRI of the shoulders and thighs demonstrated increased signal on T2 STIR imaging (Fig. 1) consistent with the diagnosis. Cardiac MRI revealed increased values on both T1 and T2 mapping (Fig. 2), suggestive of myocarditis, with no inducible ischemia or significant valvular dysfunction. The patient declined muscle biopsy. Intramuscular methylprednisolone was commenced with good clinical response.

**Figure 3:**
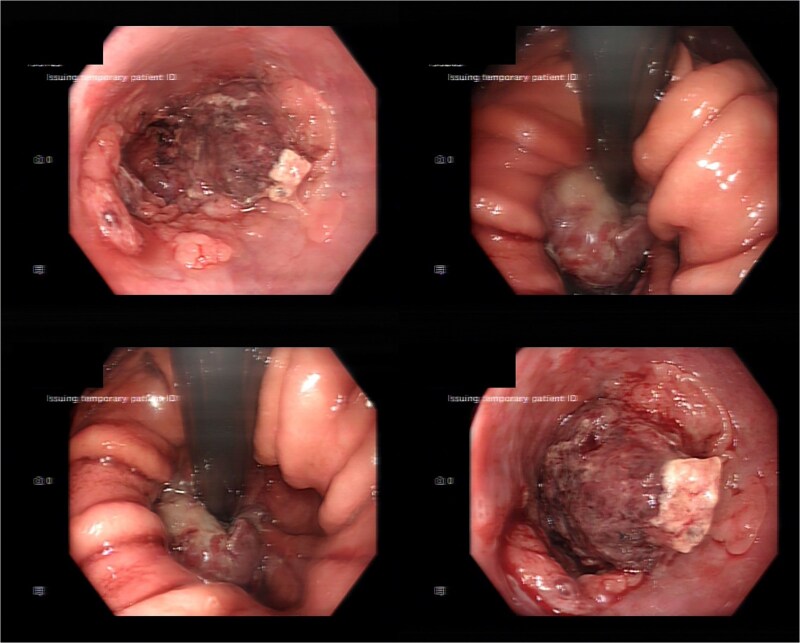
Endoscopic appearance of oesophageal tumour.

Computed tomography identified abnormal thickening of the lower oesophagus with associated lymphadenopathy. Oesophagogastroduodenoscopy revealed a circumferential tumour in the lower oesophagus (Fig. 3). Biopsy demonstrated poorly differentiated adenocarcinoma. Following multidisciplinary team review, the patient was deemed unsuitable for surgical resection or systemic therapy due to his poor performance status and the high-grade features seen on biopsy. He was referred for hospice-based palliative care.

## Discussion

We report a highly unusual case of CAM in which acute heart failure and airway compromise were the dominant features. This case highlights the diagnostic challenges associated with this highly variable condition, in which classical clinical features such as skin rashes may be transient and therefore not apparent at presentation. Dermatomyositis should be considered in all patients with muscle weakness and rash, even if the rash is not evident at the time of presentation. The recognition of dermatomyositis in this case prompted the search for malignancy, which ultimately yielded the diagnosis of oesophageal adenocarcinoma.

This case was challenging due to the unusual presenting features, absence of rash at presentation, and recent exposure to oral antibiotics and corticosteroids. The prompt administration of high dose steroids for airway compromise at presentation may have partially treated the underlying inflammatory disorder and masked the typical clinical features. The patient’s proximal myopathy, congestion and atrial arrhythmias were initially attributed to high-dose corticosteroid use. Similarly, the transient rash was initially attributed to the amoxicillin prescribed a week prior. Careful history-taking later revealed that both the myopathy and the rash had preceded the new medications, making drug reactions unlikely.

The 2017 EULAR/ACR criteria for diagnosis of IIM do not require muscle biopsy or electromyography [3]. The clinical and laboratory features in this case would yield a diagnosis of ‘definite IIM’ according to these criteria. The diagnosis was further supported by positive anti-TIF1 antibodies, which have a specificity of 92% for CAM [4], and characteristic MRI findings. While muscle biopsy and electromyography would have been helpful to support the diagnosis, the patient declined these investigations. The patient clearly described a rash consistent with the ‘V-sign’ and ‘shawl sign’ of dermatomyositis. Dermatomyositis was therefore felt to be the most likely subtype of IIM rather than related conditions including polymyositis or inclusion body myositis.

Laryngeal manifestations of dermatomyositis are extremely rare and are usually limited to inflammatory myopathy of pharyngeal musculature [5]. Airway oedema from dermatomyositis is extremely rare with only one previous case report describing this phenomenon [6]. While the precise pathophysiology remains uncertain, it is possible that increased levels of vascular endothelial growth factor in the setting of muscular inflammation could lead to increased permeability and local oedema.

The unexplained troponin T elevation in this patient in the absence of ischemic chest pain prompted testing of troponin I. Cardiac troponin I immunoassays may be less susceptible to cross-reaction with skeletal muscle troponin isoforms and can therefore be a helpful adjunct where dual cardiac and skeletal muscle pathology is suspected [7]. The discrepancy between these results triggered the search for a systemic myositis, which ultimately led to the diagnosis.

We believe that this is the first reported case of supraglottitis and acute heart failure as a presentation of paraneoplastic dermatomyositis. The subsequent identification of a high-grade oesophageal adenocarcinoma highlights the importance of appropriate malignancy screening in patients with IIM. Given the highly variable nature of the condition, dermatomyositis should be considered in the differential of all patients presenting with muscle weakness and recent history of rash, even if other symptoms dominate at the time of presentation.
